# Testing for association of the monoamine oxidase A promoter polymorphism with brain structure volumes in both autism and the fragile X syndrome

**DOI:** 10.1186/1866-1955-6-6

**Published:** 2014-03-26

**Authors:** Thomas H Wassink, Heather C Hazlett, Lea K Davis, Allan L Reiss, Joseph Piven

**Affiliations:** 1 Department of Psychiatry, University of Iowa Carver College of Medicine, 1-191 MEB, Iowa City, Iowa 52242, USA; 2 Neurodevelopmental Disorders Research Center and Department of Psychiatry, University of North Carolina, Chapel Hill, North Carolina, USA; 3 Department of Medicine, Section of Genetic Medicine, University of Chicago, Chicago, IL, USA; 4 Department of Psychiatry and Behavioral Sciences, Center for Interdisciplinary Brain Sciences Research, School of Medicine, Stanford University, Stanford, California, USA

**Keywords:** Autism, Fragile X syndrome, Brain structure, Monoamine oxidase A, Polymorphism

## Abstract

**Background:**

Autism and the fragile X syndrome (FXS) are related to each other genetically and symptomatically. A cardinal biological feature of both disorders is abnormalities of cerebral cortical brain volumes. We have previously shown that the monoamine oxidase A (MAOA) promoter polymorphism is associated with cerebral cortical volumes in children with autism, and we now sought to determine whether the association was also present in children with FXS.

**Methods:**

Participants included 47 2-year-old Caucasian boys with FXS, some of whom also had autism, as well as 34 2-year-old boys with idiopathic autism analyzed in a previous study. The MAOA promoter polymorphism was genotyped and tested for relationships with gray and white matter volumes of the cerebral cortical lobes and cerebro-spinal fluid volume of the lateral ventricles.

**Results:**

MAOA genotype effects in FXS children were the same as those previously observed in idiopathic autism: the low activity MAOA promoter polymorphism allele was associated with increased gray and white matter volumes in all cerebral lobes. The effect was most pronounced in frontal lobe gray matter and all three white matter regions: frontal gray, F = 4.39, *P* = 0.04; frontal white, F = 5.71, *P* = 0.02; temporal white, F = 4.73, *P* = 0.04; parieto-occipital white, F = 5.00, *P* = 0.03. Analysis of combined FXS and idiopathic autism samples produced *P* values for these regions <0.01 and effect sizes of approximately 0.10.

**Conclusions:**

The MAOA promoter polymorphism is similarly associated with brain structure volumes in both idiopathic autism and FXS. These data illuminate a number of important aspects of autism and FXS heritability: a genetic effect on a core biological trait of illness, the specificity/generalizability of the genetic effect, and the utility of examining individual genetic effects on the background of a single gene disorder such as FXS.

## Background

Autism is a behaviorally defined syndrome characterized by extensive phenotypic variation and etiological heterogeneity. Symptom constellations, disease severity, and associated biological traits can differ markedly from one affected individual to another. This is true even in cases attributable to single genetic defects such as chromosome 15q11-13 duplications [1] or 16p11.2 deletions [2]. Based on heritability studies, while some phenotypic variability may be due to environmental factors, a substantial portion can be attributed to heritable ‘modifying’ factors that have little effect on disease risk itself [3-5].

Identifying and studying modifying genes can be important clinically. In hypertension, for example, common variation in the renal NaCl-transporter SLC12A3 (NCCT) gene, while only minimally associated with disease, is strongly associated with biological traits related to blood pressure [6] and is also the site of action for thiazide diuretics [7]. Patterns of association in autism and other neuropsychiatric disorders appear to be similar: genes of neurotransmitter systems, while minimally associated with the disorders, are more strongly associated with important biological traits and are the sights of action of many psychiatric medications [8].

We have previously investigated relationships between genes of the serotonin system, including monoamine oxidase A (MAOA), and the associated trait of brain morphology in children with autism. Abnormalities of the serotonin system have repeatedly been described in children with autism, and MAOA is centrally involved in synaptic processing of serotonin. While association studies of MAOA variants in autism have produced uneven results [9-11], the gene has been associated with cognitive and emotional traits of relevance to the disorder [12-15], and the MAOA protein is the primary target for MAO inhibitors, an important class of psychiatric medication. We selected brain structure as an associated trait because cortical enlargement is a replicable phenotypic feature of autism thought to be intrinsically related to the disease process [16]. We found that while not associated with autism itself, a low activity allele of a functional MAOA promoter polymorphism was associated with increased cerebral cortical gray and white matter volumes in children with autism but not in typically developing children [9].

The generalizability of this result within autism spectrum disorders (ASDs) is, however, unclear. Given that autism comprises many different etiologies, we do not know whether the association we observed would characterize all cases or only certain etiologically distinct subgroups. Single gene disorders that produce the autism phenotype, such as fragile X syndrome (FXS), can be useful in elucidating the specificity of such effects. FXS is a monogenic disorder with a characteristic behavioral phenotype that often includes features of autism. Children with FXS experience, for example, social anxiety and avoidance, repetitive behaviors such as hand-flapping, and impaired speech and communication [17,18]. Up to one-third of children with FXS meet diagnostic criteria for autism, and FXS is the most common known cause of autism, accounting for 2% to 3% of cases. Children with FXS also have characteristic patterns of brain structure volumes that are similar in some respects but that also differ from those seen in autism [18-21]. Thus we tested whether an association between a genetic variant and a biological measure that was identified in idiopathic autism was also found in a single gene disorder commonly associated with autism. Results from these analyses clarify the specificity of the original MAOA finding and provide a model for investigating interaction among specific genes on biological traits of illness.

## Methods

### Participant recruitment and clinical and behavioral assessment

The study protocols were approved by the human subjects committees at the Stanford University School of Medicine and the University of North Carolina, Chapel Hill (UNC). Consent was obtained from parents.

Children with autism were drawn from an ongoing UNC longitudinal study of brain development in very young children with autism [16]. These children were primarily referred from nine specialty clinics for pervasive developmental disorders in North Carolina (Treatment and Education of Autistic and Related Communication Handicapped Children (TEACCH) centers). Subjects with autism were enrolled between 18 and 35 months of age after receiving a clinical diagnosis of an autism spectrum disorder. Medical records and developmental history were reviewed. Subjects were excluded for having evidence of a medical condition thought to be associated with autism, including FXS, tuberous sclerosis, gross central nervous system injury (for example, cerebral palsy, significant perinatal or postnatal complications or trauma, drug exposure), seizures, and significant motor or sensory impairments.

Diagnosis was confirmed using the Autism Diagnostic Interview-Revised (ADI-R) [22] and the Autism Diagnostic Observation Schedule-Generic (ADOS-G) [23]. Subjects were included if they met ADI-R algorithm criteria for autism and obtained ADOS-G scores consistent with autism. Diagnosis was reconfirmed at 4 years of age. All of the cases met DSM-IV criteria for autistic disorder. Study approval was acquired from the University of North Carolina institutional review board, and written informed consent was obtained from parents or custodial guardian for each subject. The total autism sample comprises 51 children. DNA was available for 34 boys from this sample, all of whom were Caucasian, with an average age at the time of the scan of 2.71 ± 0.30 years.

Children with FXS were recruited using Stanford and UNC registry databases, postings on the National Fragile X Foundation Web site and in their quarterly newsletter, and mailings to regional FXS organizations. Inclusion in the FXS group required DNA testing that confirmed the fragile X full mutation using the standard Southern blot technique. Exclusion criteria included preterm birth (<34 weeks gestation), low birth weight (<,2000 g), and any serious medical or neurological condition affecting growth and development (for example, seizure disorder, diabetes, or congenital heart disease). The total FXS sample also now comprises 51 children who were first imaged between the ages of 18 and 35 months. DNA was available for 47 boys from this sample, all of whom were Caucasian, with an average age at the time of the scan of 2.90 ± 0.60 years. In addition, children with FXS were evaluated for autism using the ADI-R and ADOS-G as described above; 17 FXS subjects met diagnostic criteria for autism (FXS + ASD) while 30 did not (FXS - ASD).

All children with FXS and/or autism were further assessed with a battery of measures including the Mullen Scales of Early Learning [24] , the Vineland Adaptive Behavior Scales [25], behavioral rating scales (for example, Repetitive Behavior Scales), and a standardized neurodevelopmental examination.

Lastly, we compared our results from the patient samples with those from a group of older typically developing children described in our previous report [9]. Briefly, we acquired DNA from a sample of children who had undergone an MRI scan at the University of Iowa Hospital and Clinics as part of their involvement in another study. Exclusion criteria for this group included presence of braces, major medical, neurologic, or psychiatric illness, or history of learning disability (information obtained from parents during screening process). Average full scale IQ for this group was 112 (±18). Only genotypes from boys in typically developing sample were analyzed, resulting in a cohort of 39 Caucasian boys with an average age at the time of scan of 12.5 ± 2.21 years.

### MRI acquisition

The FXS and autism participants underwent a multi-modal magnetic resonance imaging (MRI) brain scan. Participants were sedated during their MRI by a pediatric anesthesiologist who administered and monitored the sedation throughout the scan. MRI acquisition was performed on General Electric 1.5-T Signa LX scanners (GE Healthcare, Milwaukee, WI, USA) using standard transmit/receive 4-channel head coils. An identical pulse sequence protocol was used at both the UNC and Stanford sites that maximized contrast between gray matter (GM), white matter (WM), and cerebrospinal fluid (CSF) for the participants’ age range: (1) a coronal T1 IR Prepared with T1 300 ms, TR 12 ms, TE 5 ms, 20° flip angle, at 1.5 mm thickness with 1 NEX, 20 cm field of view (FOV), and a 256 × 192 matrix; and (2) a coronal PD/T2 2D dual FSE, TR 7200 ms, TE 17/75 ms, at 3.0 mm thickness with 1 NEX, 20 cm FOV, and a 256 × 160 matrix. An MRI quality control phantom was scanned after each subject at both sites to standardize assessments over sites, individuals, and time. We also performed extensive additional quality control procedures and analyses to ensure comparability of scans across the two sites, all of which are described in a previous publication [21].

The images for the typically developing adolescent sample were obtained on a 1.5 Tesla GE Signa MR scanner. Three different sequences were acquired for each subject: T1, T2, and Proton Density. Processing of the images after acquisition was done using a locally developed family of software programs called BRAINS (acronym for Brain Research: Analysis of Images, Networks, and Systems). Details of the image analysis are published elsewhere [26-30]. Briefly, The T2 and proton density images were aligned to the spatially normalized T1 image using an automated image coregistration program. A Talairach-based atlas coordinate system was overlaid onto each individual brain, aligning with anatomical landmarks of that brain without normalization to a standardized brain size [31]. These coordinates were then used to generate automated measurements of frontal, temporal, parietal, and occipital lobes, cerebellum, and subcortical regions. This method permits morphological measurements to be made in non-normalized or ‘raw’ space.

### Image processing

For all images, initial image processing to register and align the T1 and PD/T2 scans into a standardized plane was conducted with BRAINS2, which was developed at the University of Iowa [27,30]. Images were processed for tissue segmentation using an adaptation by our lab of the Expectation Maximization Segmentation (EMS) software originally developed at the Catholic University of Leuven [32]. We also developed and used a probabilistic atlas for tissue segmentation of the 2-year-old brain. The automated tissue segmentation protocol has been previously described in detail [16] and was used to generate gray and white matter volumes for the frontal, temporal, and combined parietal-occipital lobes, and for the cerebellum.

### Genotyping

For this study, we genotyped and analyzed the MAOA promoter polymorphism. PCR amplification of the polymorphism was performed according to a previously described protocol [33]. PCR products were electrophoresed on 6% polyacrylamide gels that were stained with silver and read by two independent raters with discrepancies resolved by regenotyping. The proper genotype grouping for the MAOA promoter VNTR is based on functional expression data which shows that 3 and 5 repeat variants have low enzymatic activity (MAOA-L) while 3.5 and 4 repeats show high activity (MAOA-H) [33]. As *MAOA* is an X chromosome gene and only boys were analyzed, all genotypes were hemizygous.

### Statistical analysis

Analysis of covariance (ANCOVA) was performed in SAS to test for relationships between genotypes and brain structure volumes. Because some of the brain structure volumes were not normally distributed, we used non-parametric analogs of the ANCOVAs in which all continuous variables were ranked and the rank order values were analyzed instead of the raw values. The independent predictors were *MAOA* genotype, presence or absence of FXS, and presence or absence of autism. Structure volumes were the dependent measures and covariates included age at the time of scan and the Vineland Adaptive Behavior composite score (which provided more variance than the IQ measure derived from the Mullen scale). We also tested all interaction terms, which were kept in the model only if significant.

For those brain structure volumes that were significantly influenced by MAOA genotype we calculated the percent difference between the adjusted means for the genotype groups. We also calculated eta^2^ (semi-partial eta^2^ as implemented in SAS), an effect size statistic that estimates the amount of variance in the dependents measure accounted for by the independent measure after removing effects of the covariates.

Determining appropriate significance thresholds for the statistical tests is complex. We were following up positive associations that were in a specific direction, enabling the possibility of using one-tailed tests. In addition, the regional brain structure volumes are highly correlated (rather than independent), so that a strict Bonferroni correction would be overly conservative. As a compromise, we have chosen to perform two-tailed tests describing *P* values ≤0.05 as ‘nominally’ significant, while presenting all test results and *P* values so that the reader can decide.

## Results

Table 1 shows the number of subjects, average ages, IQ (Mullen), and adaptive functioning (Vineland) in each genotype group. IQ and adaptive functioning were different across diagnostic groups (Mullen: F = 7.89, *P* = 0.0008; Vineland: F = 5.61, *P* = 0.006), with the FXS + ASD group having higher scores than the other two groups. There was no association, however, between genotype and age (F = 0.02, *P* = 0.90), IQ (F = 0.96, *P* = 0.22), or adaptive functioning (F = 3.37, *P* = 0.07).

**Table 1:** Ages and allele frequencies of study subjects

	**Idiopathic autism**		**FXS - ASD**		**FXS + ASD**	
**MAOA allele**	**High**	**Low**	**High**	**Low**	**High**	**Low**
*n*	21	13	20	10	10	7
Age, mean ± sd (years)	2.7 ± 0.4	2.8 ± 0.2	2.8 ± 0.7	2.9 ± 0.6	3.0 ± 0.6	3.0 ± 0.6
IQ	54 ± 7	52 ± 7	51 ± 4	50 ± 4	60 ± 12	54 ± 5
Adaptive behavior	61 ± 6	58 ± 6	57 ± 6	57 ± 4	68 ± 12	60 ± 3

Estimate of IQ is from the Mullen Composite Standard Scale score.

Adaptive behavior estimate is from the Vineland Adaptive Behavior Composite.

**Figure 1:**
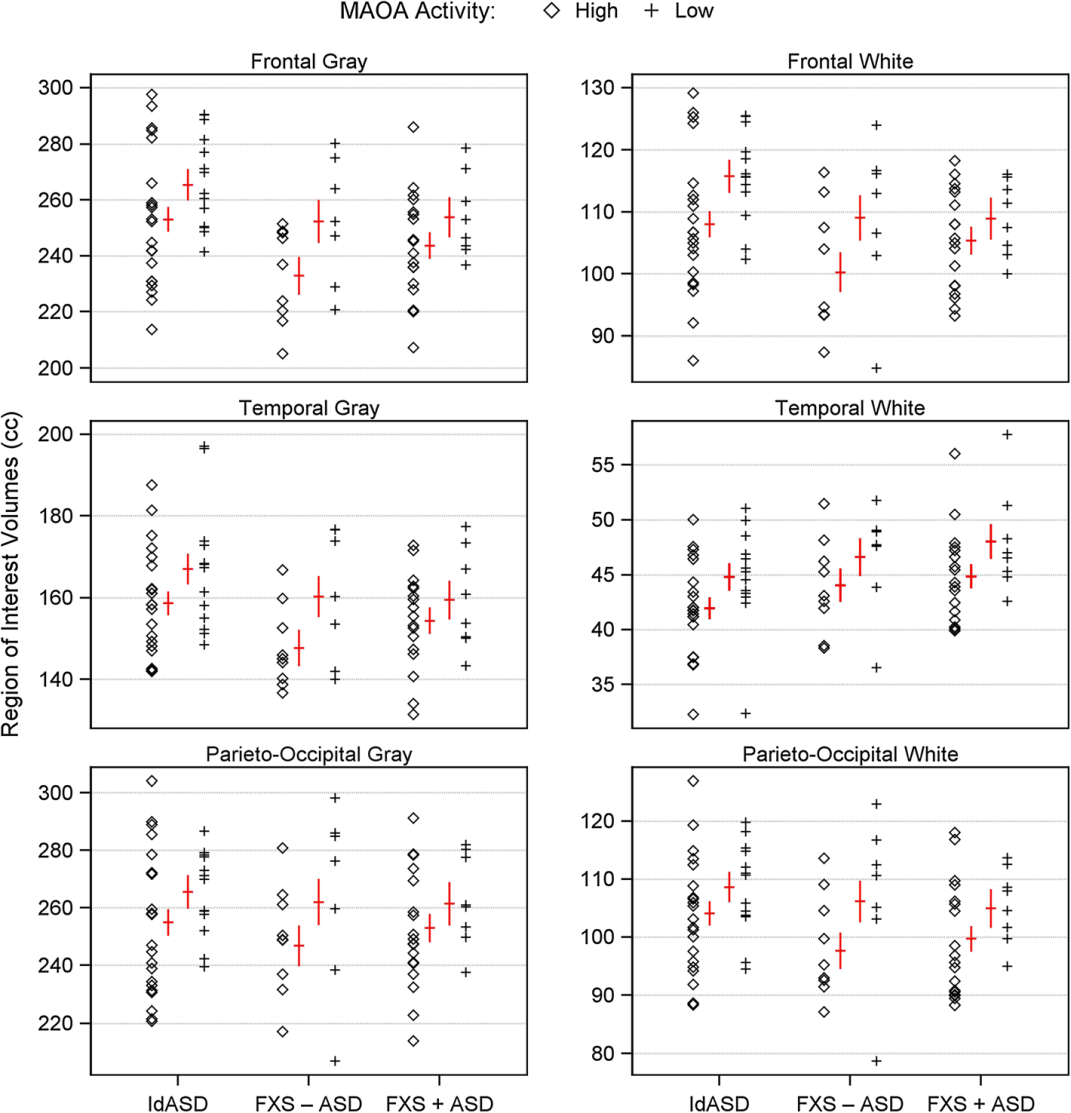
**MAOA promoter polymorphism alleles and brain structure volumes across diagnostic groups.** The figure shows gray and white matter volumes for three cerebral cortex lobes: frontal, temporal, and combined parietal and occipital. Individuals are separated according to diagnostic group: IdASD = idiopathic autism spectrum disorder, FXS-ASD = fragile X syndrome without ASD, and FXS + ASD = fragile X syndrome with ASD. Within diagnostic group, individuals are further stratified by genotype, with diamonds (◊) representing those with high activity and crosses (+) those with low activity MAOA alleles. All values have been adjusted for the covariate of age. Red crosses show least squares means (lsmeans) for each diagnosis-by-genotype group; the horizontal bar is the lsmean and the vertical is the standard error of the mean. For all three diagnostic groups across all six brain regions, the low activity allele is associated with greater volumes than the high activity allele.

Table 2 shows adjusted means of the cortical structures for the genotype groups within each diagnostic category, and Table 3 shows the ANCOVA results. MAOA genotype produced significant main effects on both gray and white matter volumes across all the cortical lobes. In all cases, the low activity allele was associated with increased volumes, an effect that was most pronounced in white matter (Figure 1). The FXS diagnostic grouping produced nominally significant effects on frontal (F = 4.46, *P* = 0.04) and temporal GM volumes (F = 3.86, *P* = 0.05): individuals with FXS had smaller volumes of these structures than individuals with idiopathic autism. There were no significant effects of the autism diagnostic grouping on cerebral cortical brain structure volumes. There were no significant genotype-by-diagnosis interactions because the effects were in the same direction in the idiopathic autism, FXS - ASD, and FXS + ASD groups and were of generally equal strength across the groups. Subjects with low activity alleles had, on average, 3.5% to 6.7% greater volumes than subjects with high activity alleles, and MAOA genotype accounted for approximately 10% to 15% of the variability in cortical lobe gray and white matter volumes after removing effects of the covariates.

**Table 2:** Brain structure volumes by diagnosis and genotype

	**All subjects**		**Autism only**		**FXS - ASD**		**FXS + ASD**	
	**High**	**Low**	**High**	**Low**	**High**	**Low**	**High**	**Low**
	**(*n*** **= 51)**	**(*n*** **= 30)**	**(*n*** **= 21)**	**(*n*** **= 13)**	**(*n*** **= 20)**	**(*n*** **= 10)**	**(*n*** **= 10)**	**(*n*** **= 7)**
Total brain volume	1261 ± 96	1330 ± 99	1271 ± 100	1334 ± 88	1246 ± 111	1338 ± 137	1254 ± 88	1315 ± 86
Frontal gray	246 ± 24	261 ± 18	251 ± 24	266 ± 16	238 ± 25	258 ± 23	243 ± 23	255 ± 16
Frontal white	105 ± 11	114 ± 11	107 ± 11	116 ± 8.2	104 ± 15	113 ± 16	104 ± 11	110 ± 8.8
Temporal gray	155 ± 13	165 ± 15	157 ± 13	168 ± 16	151 ± 14	163 ± 17	153 ± 14	162 ± 12
Temporal white	43.4 ± 4.7	46.8 ± 5.5	41.5 ± 3.6	45 ± 4.9	45.2 ± 5.5	47.9 ± 6.3	44.5 ± 4.9	50.9 ± 5.5
Parietal-occipital gray	252 ± 22	265 ± 20	253 ± 25	266 ± 15	249 ± 18	266 ± 32	251 ± 20	262 ± 16
Parietal-occipital white	101 ± 10	108 ± 9.3	103 ± 10	109 ± 8	99 ± 8.9	108 ± 14	99 ± 9.5	106 ± 6.5

Values are adjusted means ± standard deviations in cubic centimeters.

**Table 3:** Analysis of covariance results

	**MAOA genotype F tests**								
	**All patients**			**Autism**			**FXS**		
	**F**	***P***	**eta**^**2**^	**F**	***P***	**eta**^**2**^	**F**	***P***	**eta**^**2**^
Total brain volume	8.51	0.005	0.10	3.66	0.07	--	4.24	0.05	0.09
Frontal gray	7.77	0.007	0.10	3.64	0.07	--	4.39	0.04	0.09
Frontal white	15.46	0.0002	0.16	10.58	0.003	0.25	5.71	0.02	0.09
Temporal gray	5.33	0.02	0.07	2.43	0.13	--	2.00	0.17	--
Temporal white	13.29	0.0005	0.14	13.84	0.0008	0.30	4.73	0.04	0.08
Parietal-occipital gray	6.72	0.01	0.09	2.71	0.11	--	2.92	0.10	--
Parietal-occipital white	9.89	0.003	0.12	4.61	0.04	0.12	5.00	0.03	0.11

**Figure 2:**
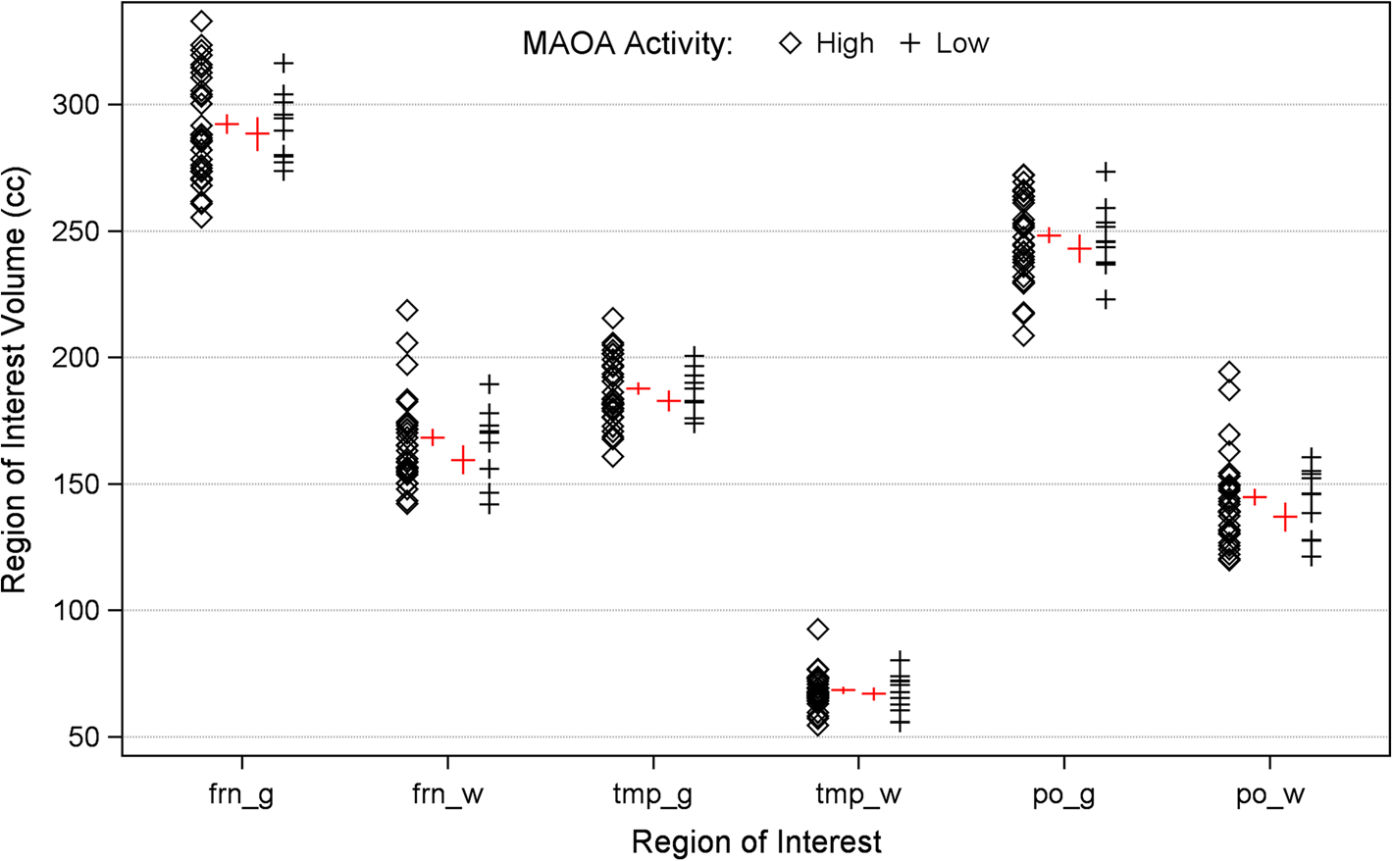
**MAOA promoter polymorphism alleles and brain structure volumes in typically developing control children.** For purposes of comparison with Figure 1, this figure shows gray and white matter volumes for the three cortical lobes in typically developing children. Values have been adjusted for age. In contrast to the children with autism and/or FXS, MAOA promoter polymorphism genotype is not associated with different brain structure volumes in these children.

As previously described, 28 typically developing children had the high activity allele and 11 the low activity allele. There were no significant differences in these children for any brain structure volume based on MAOA genotype (Figure 2).

## Discussion

We have previously shown that a functional promoter polymorphism of the MAOA gene is associated with brain structure volumes in children with idiopathic autism [9]. Given the phenotypic overlap with autism, we now tested for a similar association in children with the fragile X syndrome (FXS). We found, as in idiopathic autism, that the low-expressing allele was associated with increased cerebral cortical gray and white matter volumes in the FXS children, both those with and without autism (Figure 1). These associations were not observed in older typically developing children assessed in our previous study (Figure 2). We thus demonstrate that this modifying genetic effect on an endophenotypic trait is found in two neurodevelopmental disorders, and we demonstrate the utility of FXS and autism as a system for testing relationships amongst specific genes within the heterogeneity of ASDs.

The plausibility of the MAOA polymorphism producing these brain effects is supported by multiple lines of research. Serotonin influences numerous aspects of brain development, as well as ongoing synaptic activity throughout life [34,35]. Perturbations of the serotonin system have been implicated in a variety of neuropsychiatric disorders, with particularly compelling effects in autism [36-40]. MAOA is the primary enzyme responsible for degrading synaptic monoamine neurotransmitters, including serotonin, and levels of these neurotransmitters in the brain vary in association with levels of the MAOA protein [41-44].

Alterations in expression of MAOA are, in turn, associated with neuropsychiatric disorders. Two null mutations cause severe phenotypes. Norrie’s disease is characterized by mental retardation, autistic behavior, and motor hyperactivity, and is caused by an X-chromosomal deletion that includes MAOA [45,46], while Brunner’s syndrome includes violent and criminal behavior in the phenotype and is due to an MAOA stop mutation [47,48]. The MAOA promoter polymorphism, by contrast, is associated with more moderate expression effects. The polymorphism is a 30 base pair VNTR that is 1.2 kb upstream of exon 1 [49]. When present as 3.5 or 4 copies, the repeat is associated with increased MAOA expression, while 2, 3 or 5 copies are associated with decreased expression [33,50]. The repeat has also been associated with numerous less severe but more common neuropsychiatric phenotypes [51-54]. Of relevance to autism, the low activity allele has been associated with increased severity of a range of social and behavioral difficulties, including sensory behaviors, arousal regulation, aggression, social communication skills [54,55], a lower IQ [54], and, through our previous work, cerebral cortical enlargement [9]. This association with brain structure is noteworthy because increased head circumference and enlargement of the cerebral cortex are highly replicable biological correlates of autism [16,56].

The current findings are consistent with a more general relationship between serotonin and brain structure suggested by our research. In addition to MAOA, we have also tested a functional promoter polymorphism of the serotonin transporter gene (SERT) [57]. Just as with MAOA, we found that an allele associated with decreased expression of SERT is associated with increased cerebral cortical gray and white matter volumes in idiopathic autism. For both genes, decreased expression is associated with increased synaptic and central nervous system (CNS) serotonin levels [41,42,58]. Thus a picture begins to emerge of serotonin system genetic variation influencing brain structure where increased serotonin expression is associated with increased cerebral cortical volumes in children with neurodevelopmental disorders, particularly autism and FXS.

The findings also show how studying single gene disorders can help to clarify the specificity of genetic effects found in more complex, heterogeneous disorders. Autism has a highly variable phenotype, suggesting an array of interacting genetic factors that influence the expression of disease. FXS is a monogenic disorder in which some children have phenotypic features of autism while others do not. Through our examination, we find that the MAOA polymorphism produces the same effects on brain structure in three groups of affected children: those with idiopathic autism, those with FXS and autism, and those with FXS but no autism. We do not find any effect of the polymorphism on brain structure in typically developing children, though we do note that this is not an ideal comparison group because they are older than the patients and their images were acquired through a different scan protocol.

Furthermore, though an understanding of why the MAOA polymorphism would produce brain effects in some children but not others is not known, studies of mice support this type of interaction. Mice of different genetic backgrounds display markedly different patterns of social behavior, with some strains exhibiting social deficits resembling those seen in autism [59,60]. The same genetic defect placed on these different backgrounds can produce strikingly different phenotypes. Moy et al., for example, reported that targeted disruption of Fmr1 produced sociability deficits in FVB/129 mice but not in C57BL/6 J mice [61]. More closely related to our study, Page et al. used mice to examine interactive effects of two autism-related genes on brain structure [62]. Mice haploinsufficient for either the SERT or PTEN gene had enlarged brains as well as deficits in social interaction. After crossing, mice haploinsufficient for both genes had more substantial brain enlargement and more severe sociability deficits.

## Conclusion

In our study examining two gene variants (the MAOA polymorphism and the FXS anomaly) and brain volume in humans, we do not find evidence of a combinatorial effect, additive or otherwise. Rather, we find that the association of MAOA with brain structure in similar in children with either FXS or autism or both. The relationship does not generalize to older typically developing children; testing in additional disorders will further clarify the boundaries of the association.

## Competing interests

The authors declare that they have no competing interests.

## Authors’ contributions

TW performed data analysis and manuscript writing. HH performed data collection and manuscript review. LD carried out genotyping, data analysis, and manuscript review. PN performed data collection and manuscript review. AR performed data collection and manuscript review. JP performed data contribution, manuscript writing, and manuscript review. TW had full access to all of the data in the study and takes responsibility for the integrity of the data and the accuracy of the data analysis. All authors read and approved the final manuscript.
